# Editorial: Functional biomaterials for drug delivery

**DOI:** 10.3389/fbioe.2025.1550444

**Published:** 2025-02-12

**Authors:** Yi Chen, Zhengwei Cai

**Affiliations:** Department of Orthopaedics, Shanghai Key Laboratory for Prevention and Treatment of Bone and Joint Diseases, Shanghai Institute of Traumatology and Orthopaedics, Ruijin Hospital, Shanghai Jiao Tong University School of Medicine, Shanghai, China

**Keywords:** drug delivery, functional biomaterials, responsive materials, nanotechnology, AI-assisted material design, electrospun nanofibers

## Introduction

Drug delivery is a major interest in medicine, but it is still severely limited by a multitude of biological barriers such as enzyme degradation and difficulties in transporting to cells. These challenges have inspired the development of functional biomaterials that can dynamically manipulate their interactions with the environment, ultimately improving drug stability, targetability, and therapeutic effectiveness.

This Research Topic focuses on recent progress highlighting the critical role of functional biomaterials in advancing drug delivery systems. The studies are categorized into three main approaches: (1) the development of intelligent biomaterials for targeted drug release, (2) the application of nanotechnology to improve drug stability and bioavailability, and (3) the advancement of delivery systems to overcome the limitations of classical treatments in delivering therapeutic agents for complex diseases.

## Intelligent responsive materials

Smart responsive materials for drug delivery systems include a thermosensitive hydrogel system using chitosan, erythropoietin (EPO), and FK506, developed by Gu et al. This is a fast-gelling hydrogel at body temperature, and this property is useful in irregularly shaped periodontal tissue defects. It allows the controlled release of encapsulated drugs whilst forming a microenvironment that is beneficial for tissue regeneration. In their rat experimental periodontitis model, Zhang et al. found that hydrogel decreased inflammatory cytokines (TNF-α, IL-6, and IL-1β) and upregulated Collagen I, Runx2, OPN, and OCN to promote bone regeneration. In addition to its high porosity, the hydrogel enhances cell penetration and nutrient exchange, thus serving as an excellent local delivery platform for periodontal regeneration.

Oliveira et al. developed a polyvinyl alcohol-carboxymethylcellulose (PVA-CMC)-based hydrogel system embedded with natural bioactive extracts of dragon’s blood resin and sage. Dragon’s blood, containing phenolic compounds like gallic acid and catechin, exhibits anti-inflammatory and antioxidant effects, enabling it to dynamically respond to wound microenvironments by accelerating collagen formation and epithelial regeneration. Sage extract, with bioactive molecules like thujone and cineole, responds to inflammatory signals by regulating cytokines and enhancing angiogenesis for tissue repair. The hydrogel’s ability to absorb liquids and release bioactives in a controlled manner aligns with the dynamic demands of wound microenvironments, showcasing its potential as a plant-based solution for effective wound healing.

## Nanotechnology in drug delivery

Nanotechnology has opened up exciting possibilities for precision drug delivery. Jiao et al. reviewed non-viral vectors, highlighting their advantages in safety and lower immunogenicity. These vectors include polymers, liposomes, and lipid nanoparticles, which are engineered with surface modifications to improve gene delivery efficiency and reduce immune responses. For example, polymer-based nanocarriers like polyethyleneimine (PEI) utilize the “proton sponge” effect to enhance endosomal escape, while liposomes benefit from high biocompatibility and surface functionalization to target vascular endothelial cells in cardiovascular diseases. These innovations offer promising directions for gene therapy, although challenges such as precise nucleic acid delivery and long-term efficacy remain.

Nanotechnology Development of Oocyte Cryopreservation Poly(Lactic-co-Glycolic Acid)-Resveratrol (PLGA-RES) Nanocomposite by Hai et al. combines PLGA’s biodegradability with the potent antioxidant properties of RES. This innovative approach helps combat oxidative stress occurring during frozen and thaw cycles. Moreover, the nanocomposite showed much higher oocyte viability and maturation, overcoming a major challenge encountered in the field of reproductive medicine. This study demonstrates the significant potential of engineered nanoscale biomaterials to protect against physicochemical damage at the cellular level under severe environmental conditions.

## Advanced delivery systems for complex diseases

Innovative delivery systems with multiple functions have become key platforms for confronting complex diseases. A motile hydrogel microrobot for osteosarcoma treatment via a magnetically propelled method was fabricated by Wang et al. Under an external magnetic field, this microrobot penetrates into the tumor site to co-deliver MET inhibitor SCR1481B1 and anticancer drug Anlotinib. The hydrogel matrix is also capable of providing the sustained release of the drugs, which is favorable for the therapeutic effect since it allows higher retention of the drug at the tumor site. Preclinical studies showed excellent antitumor activity of the system in both 2D and 3D tumor models without significant toxicity to healthy tissues.

Using electrospun nanofibers to promote diabetic wound healing was discussed by Jiang et al. This review highlights the applications of electrospun nanofiber scaffolds in promoting diabetic wound healing, focusing on their high surface area, tunable porosity, and biocompatibility, which make them effective localized drug delivery systems and structural supports. By incorporating various therapeutic agents, these scaffolds have demonstrated the ability to modulate inflammation and facilitate granulation tissue formation, contributing to the wound healing process in diabetic patients.

Exosome-based therapies have emerged as a promising candidate for treating orthopedic degenerative diseases. In a review by Yue et al., the authors provided a comprehensive analysis of exosome applications, highlighting their role as carriers for intercellular communication and their potential in regenerative medicine. Exosomes have been shown to facilitate cartilage and bone repair by delivering bioactive substances such as proteins and nucleic acids. The review also discussed strategies to address challenges such as rapid clearance and limited retention of exosomes *in vivo*, including engineering modifications and the use of biomaterials to improve their stability and targeting efficacy in therapeutic applications.

## Future perspectives in functional biomaterials

Altogether, studies included in this Research Topic showcase the variety of functional biomaterials and their role in drug delivery. These studies focus on addressing the challenges of complex diseases by leveraging advancements in materials science and biomedical engineering, contributing to preparations for future clinical applications.

Future research in functional biomaterials aims to bridge the gap between laboratory studies and clinical applications. Emerging strategies, such as AI-assisted material design, hold potential for optimizing biomaterial properties to meet specific therapeutic needs. Additionally, investigating interactions between biomaterials and the immune system is critical to ensure safety and efficacy. Furthermore, expanding the application of innovative platforms like microrobots and nanofibers to diverse disease contexts will further advance their role in personalized medicine.

